# Measuring blood–brain barrier dysfunction: a critical appraisal of fluid biomarkers, in vitro models, in vivo imaging, and *post‐mortem* approaches

**DOI:** 10.1002/alz.71263

**Published:** 2026-03-15

**Authors:** Shakira A. van der Panne, Helena Durrant, Jannis F. Heuer, Ivana Kancheva, Arno Stellingwerf, Lieuwe T. K. M. Verkaar, Damon Verstappen, Pascalle Mossel, Walter H. Backes, Erik N. T. P. Bakker, Jurgen A. H. R. Claassen, Marcel M. Verbeek, Louise van der Weerd, Matthias J. P. van Osch, Helga E. de Vries

**Affiliations:** ^1^ Department of Biomedical Engineering and Physics Amsterdam University Medical Centre Location AMC Amsterdam The Netherlands; ^2^ Amsterdam Neuroscience Amsterdam University Medical Centre Amsterdam The Netherlands; ^3^ Amsterdam Cardiovascular Sciences Amsterdam University Medical Centre Amsterdam The Netherland; ^4^ C.J. Gorter MRI Center Department of Radiology Leiden University Medical Center Leiden The Netherlands; ^5^ Department of Molecular Cell Biology and Immunology Amsterdam University Medical Centre Location VUmc Amsterdam The Netherlands; ^6^ Department of Neurology Donders Institute for Brain Cognition and Behaviour Radboud University Medical Centre Nijmegen The Netherlands; ^7^ Radboud Alzheimer Centre Radboud University Medical Centre Nijmegen The Netherlands; ^8^ Department of Geriatric Medicine Radboud University Medical Centre Nijmegen The Netherlands; ^9^ Department of Radiology & Nuclear Medicine Maastricht University Medical Centre Maastricht The Netherlands; ^10^ Mental Health and Neuroscience Research Institute (MHeNs) Maastricht University Maastricht The Netherlands; ^11^ Department of Radiology Leiden University Medical Centre Leiden The Netherlands; ^12^ Cardiovascular Research Institute Maastricht (CARIM) Maastricht University Maastricht The Netherlands; ^13^ Department of Cardiovascular Sciences University of Leicester Leicester UK; ^14^ Department of Human Genetics Radboud University Medical Centre Nijmegen The Netherlands; ^15^ Department of Human Genetics Leiden University Medical Centre Leiden The Netherlands

**Keywords:** BBB dysfunction, blood–brain barrier, brain endothelium, critical appraisal, imaging, methods, review

## Abstract

The blood–brain barrier (BBB) maintains central nervous system homeostasis by regulating molecular exchange between blood and brain. BBB dysfunction is associated with aging and neurological disorders such as Alzheimer's disease, stroke, and multiple sclerosis. Diverse approaches are used to study BBB structure and function, including cell‐based models, imaging techniques, and fluid biomarkers. While each method has distinct strengths, inherent limitations complicate interpretation and limit comparability across studies. In addition, many methods require specialized expertise, hindering the interdisciplinary integration of findings. This review outlines commonly used methods to assess BBB dysfunction and critically evaluates their relevance, advantages, and drawbacks. It provides guidance for selecting suitable techniques, proposes guidelines, and highlights key challenges in data interpretation. Finally, the review emphasizes the need to clearly define the specific BBB aspect under investigation, calls for standardized protocols, and encourages combining approaches to improve research quality and translation into clinically meaningful insights and applications.

## Highlights

- BBB dysfunction is associated with aging and various neurological conditions including Alzheimer's disease, stroke, and multiple sclerosis.
- Researchers employ multiple methods to assess BBB structure and function, including cell‐based models, imaging, and fluid biomarkers.
- Each method has specific strengths and limitations that must be understood for accurate interpretation of results.
- Standardized protocols and clear definition of the BBB aspect being measured improves data quality and comparability.
- Integrating complementary approaches enhances mechanistic understanding and supports translation to clinically meaningful insights.

## 1 INTRODUCTION

The blood–brain barrier (BBB) is a dynamic bidirectional interface between the peripheral circulation and the central nervous system (CNS). It regulates the passage of molecules, ions, nutrients, and cells into and out of the brain parenchyma while actively limiting the entry of harmful substances like toxins and pathogens, thereby safeguarding CNS homeostasis.^1^, ^2^, ^3^, ^4^ Given its central role in brain function and neurological disease, accurate assessment of BBB alterations is critical for developing diagnostics and therapies.

**FIGURE 1:**
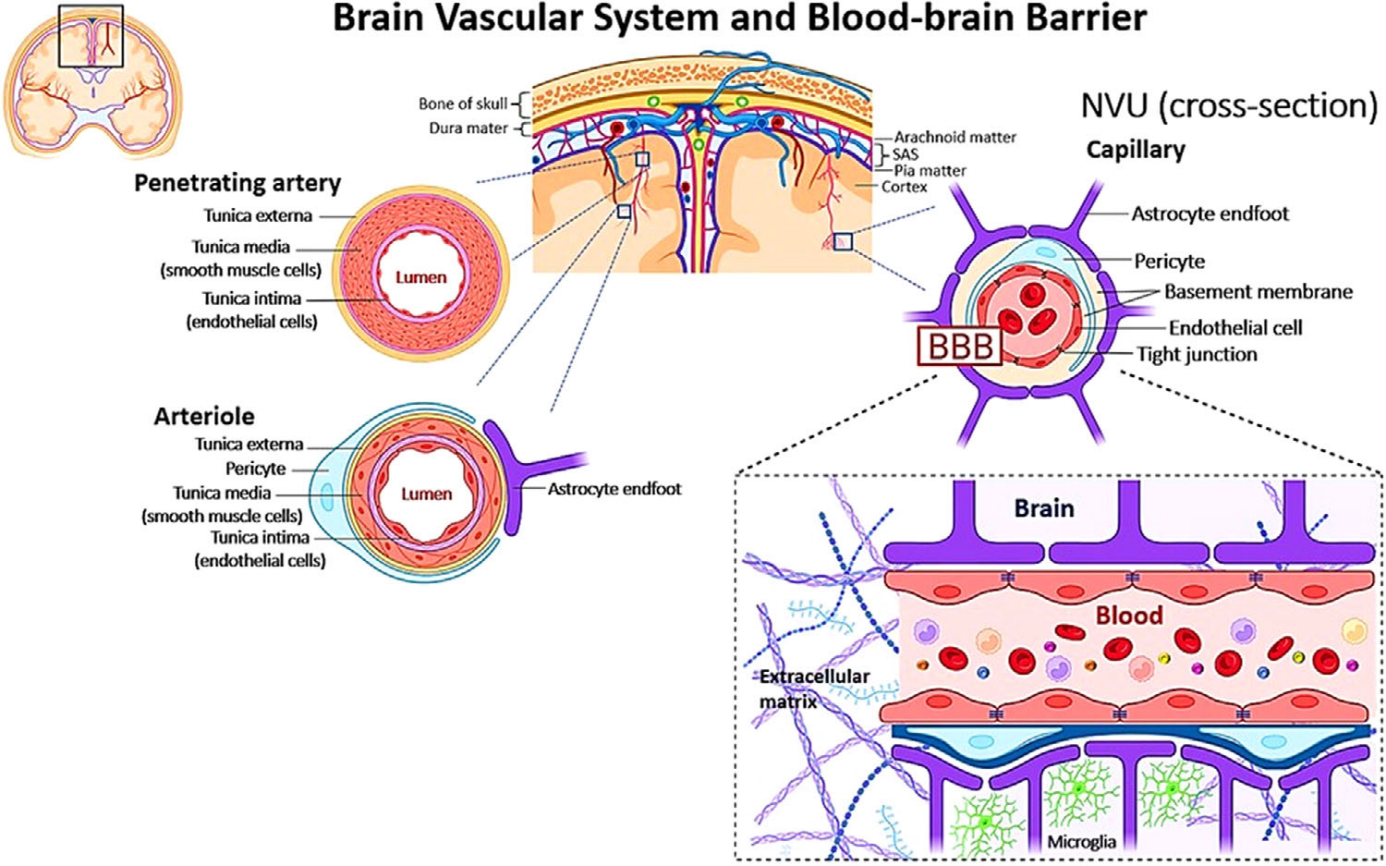
Schematic depiction of blood–brain barrier (BBB) as part of brain's vascular system. Surface pial arteries composed of multiple concentric layers of vascular smooth muscle cells branch into penetrating arteries characterized by a single smooth muscle cell layer and pericytes that wrap around the vessel. These vessels further transition into arterioles and brain capillaries where smooth muscle cells are absent and the vascular wall consists primarily of endothelial cells (ECs) and pericytes, marking the anatomical level at which the BBB is established. At the level of the BBB, the opposing membranes of EC are connected via tight junctions (TJs) expressing low paracellular and transcellular permeability. Pericytes are attached to the abluminal surface of the EC layer and are embedded in the basement membrane, which is continuous with the plasma membrane of astrocytic endfeet. The microvascular endothelium interacts with neural tissue, microglia, immune cells, and the surrounding extracellular environment, thereby regulating numerous processes with important functions in the central nervous system, including maintenance of brain‐barrier integrity, cerebral blood flow, and clearance of solutes from the parenchyma. NVU, neurovascular unit; SAS, subarachnoid space. Figure created using Biorender.com.

The BBB is primarily composed of brain endothelial cells (ECs) that line cerebral capillaries, supported by mural cells embedded in the vascular basement membrane (BM) that stabilize the vasculature. The ECs of the BBB differ from peripheral ECs by the absence of fenestrations,^5^ the presence of complex junctional complexes including tight junctions (TJs) and adherens junctions (AJs),^6^ and limited pinocytic vesicular transport.^3^, ^7^ They express two major transporter types: efflux transporters, such as P‐glycoprotein (P‐gp) that drive removal from unwanted lipophilic compounds from the brain, and nutrient transporters that supply glucose, amino acids, and other essential molecules to the brain.^8^, ^9^, ^10^ These properties tightly regulate molecular traffic, restricting para‐ and transcellular influx of compounds while allowing small lipophilic molecules and nutrients to diffuse along concentration gradients.^3^, ^11^, ^12^, ^13^, ^14^, ^15^, ^16^ CNS ECs also support receptor‐mediated clearance of waste products from brain to blood, including amyloid beta (Aβ) aggregates implicated in neurodegenerative diseases.^17^ Beyond ECs, the BBB includes the capillary BM, astrocyte endfeet that ensheath the vessel, and pericytes within the BM. These elements combined constitute an important part of the neurovascular unit (NVU), a concept describing the dynamic interplay between BBB‐related cells and the neurons and microglia in the brain tissue (Figure 1).^1^, ^3^, ^4^ Their coordinated interaction regulates critical brain functions, including metabolite exchange, neurovascular coupling, and cerebral blood flow (CBF).^11^, ^18^, ^19^

Another barrier contributing to CNS homeostasis is the blood–cerebrospinal fluid barrier (BCSFB), which is located at the epithelium of the choroid plexus (CP) rather than the parenchymal endothelium. Similar to the BBB, the BCSFB expresses various receptors, transporters, and enzymes, controlling the influx and efflux of solutes, water, nutrients, and metabolic waste.^20^, ^21^, ^22^ However, the two barriers differ in structure and function as the BCSFB features fenestrated, highly permeable capillaries that support cerebrospinal fluid (CSF) production and solute clearance via CSF turnover.^22^, ^23^, ^24^

Disruption of the structural and physiological integrity of the BBB occurs in normal aging and also contributes to multiple neurological diseases.^25^ BBB dysregulation has been reported in healthy older individuals,^25^ stroke,^26^, ^27^ Alzheimer's disease (AD), Parkinson's disease (PD),^28^, ^29^ and multiple sclerosis (MS).^30^ Compromise of barrier function leads to ion and fluid imbalance, pericyte and astrocyte dysfunction, and extravasation of plasma proteins and immune cells, driving neuroinflammation, neuronal damage, and neurodegeneration.^1^, ^2^, ^31^, ^32^ TJ disruption and increased permeability are hallmarks of ischemic and haemorrhagic stroke^26^, ^27^, ^33^ and strongly correlate with cognitive decline and dementia.^28^, ^34^, ^35^, ^36^, ^37^ BBB dysfunction can be transient or chronic and is often accompanied by altered transporter or enzyme activity that exacerbates CNS injury.^11^, ^38^, ^39^ Pathologically increased permeability may result from acute insults such as traumatic brain injury^40^ and ischemic stroke^27^ or even precede clinical symptoms as in MS.^41^ In disorders like AD, it remains unclear whether BBB dysfunction is a cause or a consequence,^42^ underscoring the need for deeper mechanistic insights.

A major challenge in BBB research is the lack of consensus on how to define BBB dysfunction. Definitions often focus either on molecular or structural changes or on impaired function of the BBB. Here, we would like to propose a clear definition of BBB dysfunction as changes in permeability via any of the BBB's transport routes, emphasizing its selective barrier function. Permeability describes the passage of molecules through the BBB, either from brain to blood, usually referred to as brain clearance, or from blood to brain, described as leakage. Structural alterations, including reduced pericyte coverage and loss of TJs and transporters, relate to functional impairment but cannot be considered as standalone BBB dysfunction as they do not reflect BBB permeability directly. However, such structural changes are frequently the focus of preclinical or *postmortem* approaches to study BBB function and are presented as surrogates of leakiness or impaired brain clearance.

**FIGURE 2:**
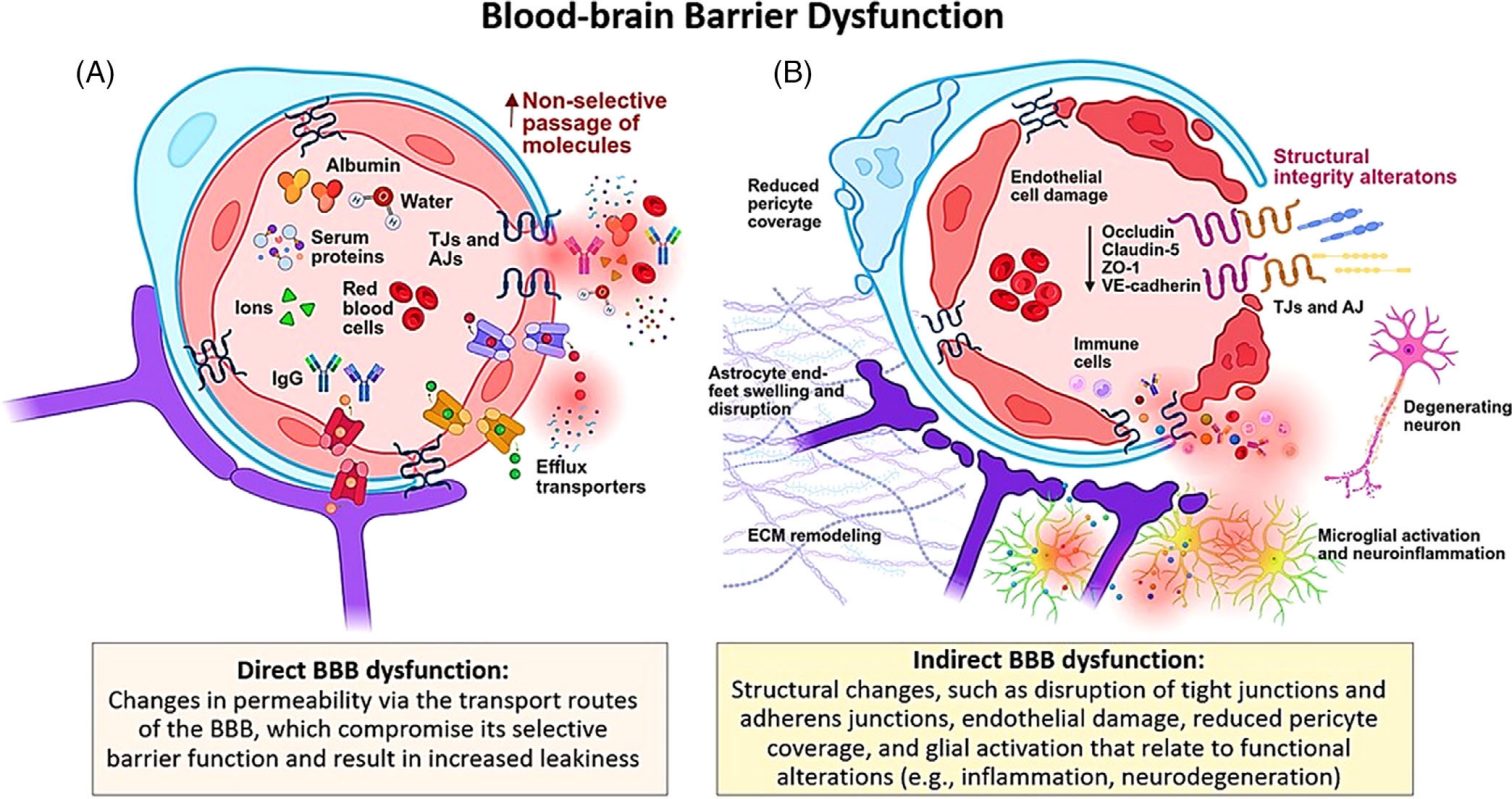
Schematic overview of blood–brain barrier (BBB) dysfunction. (A) Direct BBB dysfunction entails higher permeability of BBB to water, macromolecules, and blood‐derived proteins (e.g., albumin, immunoglobulins), which extravasate into the tissue non‐selectively. These changes are linked to structural BBB alterations (B), including disruption of TJ and AJ proteins (e.g., occludin, claudin‐5, ZO‐1, and VE‐cadherin), as well as loss of pericyte coverage and swelling of astrocytic endfeet. These processes reflect indirect BBB dysfunction and may trigger secondary changes, such as microglial activation and neuroinflammation, and over time contribute to extracellular matrix remodeling and neuronal degeneration. AJ, adherens junction; IgG, immunoglobulin G; TJ, tight junction; ZO‐1, zonula occludens 1. Figure created using Biorender.com.

In this review, techniques assessing permeability and leakage are considered direct measures of BBB dysfunction, while those evaluating structural and molecular changes to the BBB and NVU are classified as indirect. Processes such as neuroinflammation, metabolic alterations, or extracellular matrix (ECM) remodeling have a bidirectional link to BBB permeability as they influence BBB function but are also consequences of BBB impairment. As such, they do not constitute inherent BBB functions and are therefore considered secondary effects reflective of indirect BBB dysfunction. Figure 2 summarizes direct and indirect changes associated with BBB dysfunction.

Many techniques have been used to study BBB dysfunction, including fluid biomarkers, in vitro models, in vivo imaging, and *post‐mortem* analyses. Although these methods have advanced our understanding of BBB biology, there is no consensus regarding which approaches are most suitable for studying BBB dysfunction in humans, nor which specific aspects of the BBB they measure. Many commonly used methodologies rely on surrogate markers of BBB dysfunction.^43^, ^44^ Human studies remain limited and often yield inconsistent results, likely due to methodological differences and varied targets across the BBB's structural and functional domains. This is particularly challenging when translating findings from animal models to humans, and inconsistent measurement techniques may obscure mechanisms or hinder therapeutic development.^1^, ^2^, ^4^, ^45^ Despite recent advances, a critical appraisal of BBB assessment methods is needed to improve our understanding and ensure accurate, translatable measurements. This review provides such an evaluation, focusing on widely used techniques with mechanistic or clinical relevance, highlighting methodological limitations, and promoting harmonization and standardization in both research and clinical contexts.

## 2 BBB DYSFUNCTION BIOMARKERS

### 2.1 Selection guidelines for BBB dysfunction biomarkers

Despite extensive research, no standardized framework exists for selecting or evaluating biomarkers of BBB dysfunction. Different detection methods probe distinct aspects of BBB structure or function, each with specific strengths and limitations. We therefore propose the following guidelines to support the development and evaluation of BBB biomarkers.

First of all, a BBB dysfunction biomarker should demonstrate clear mechanistic and functional relevance to the disease process, reflecting one or more specific roles of the BBB (criterion 1).^46^, ^47^, ^48^, ^49^ The functional relationship between the biomarker and BBB integrity or function should be explicitly described. Second, the selected biomarker should be specific to the BBB and the disease process, originating from BBB cellular components or reflect a distinct BBB function (criterion 2).^50^ Potential BBB biomarkers should preferably not originate from cellular or functional sources that are also involved in other processes besides the BBB (e.g., reflecting glial or neuronal populations or function). As such, biomarkers should be minimally influenced by non‐BBB processes, such as traumatic‐head injury or hypertension,^50^, ^51^ as well as by variation in factors including age and sex. Relevant confounders differ between biomarker techniques and should be identified and controlled (criterion 3). When unavoidable, confounders should be addressed by minimizing differences between study conditions and applying appropriate statistical corrections.

Biomarker profiles should take into account the timing and duration of BBB dysfunction. Acute, semi‐acute, or chronic BBB dysfunction may be reflected by different biomarker levels or mechanistic responses.^52^ Such temporal considerations should be provided (criterion 4).

Finally, a novel biomarker method should be validated against independent approaches (criterion 5). Correlation with other BBB dysfunction biomarkers strengthens reliability. Preferably, the biomarker findings are replicated in independent studies. To promote replication and validation, the methods, internal standards, internal validation, quality controls, and control conditions should be well described.

### 2.2 Fluid‐based biomarkers of BBB dysfunction

Fluid biomarkers are objectively quantifiable molecules or cellular components in bodily fluids that help distinguish between healthy and pathological conditions, supporting early diagnosis, disease monitoring, and evaluation of interventions.^53^ Blood and CSF are the most commonly used matrices to assess BBB status. Biomarker targets vary widely in composition, including metabolites, miRNA, mRNA, lipids, peptides, proteins, secreted vesicles, and cells.^54^, ^55^ However, BBB fluid biomarkers are predominantly proteins or peptides, and this review focuses on these biomarkers and their detection methods.

#### 2.2.1 Fluid biomarker detection methods

Techniques for detecting BBB‐related fluid biomarkers can be broadly divided into targeted and untargeted approaches.^53^ Targeted assays investigate a predefined single analyte or panel of analytes and are typically hypothesis‐driven. These methods commonly rely on antibody affinity and include enzyme‐linked immunosorbent assays (ELISA) and derived assays such as ELLA, Single Molecule Array, and Western blotting. In contrast, untargeted approaches are primarily used for biomarker discovery, as they generate broad protein or peptide profiles with relative abundance. Common examples include mass spectrometry‐based proteomics and protein array platforms such as proximity extension assays.

Rather than detailing these well‐established techniques, this section focuses on their application in the context of the proposed biomarker selection guidelines and provides representative examples of BBB biomarkers. A more comprehensive overview of commonly used BBB biomarkers is provided in Table 1.

**TABLE 1:** Overview of most commonly proposed fluid BBB dysfunction biomarkers and their adherence to evaluation criteria.

Type	Gene	Protein	Fulfills criteria	Body fluid	Expression location	Describes	Level of establishment	Level of confidence as BBB biomarker	Indirect/direct BBB dysfunction biomarker	Specificity for BBB dysfunction	Remarks	References
R	ALB	Albumin	5*	CSF:serum	Hepatocytes	Leakage	+	−	Direct	−	Albumin ratio between CSF over serum. CSF albumin concentration could be influenced by CSF rate of reabsorbance, diffusion, and bulk flow. Albumin can also traverse the BCSFB; does not correlate with DCE‐MRI. Inconsistent results	^62^, ^63^, ^70^, ^76^, ^77^, ^78^, ^79^, ^80^
C	ANGPT‐2	Angiopoietin‐2	1,2	CSF	Endothelial and CP	Endothelial dysfunction	+/−	−	Indirect	+/−	Dual function of Ang2 probably requires investigation of Tie‐1 simultaneously. In rat CP epithelial expression described	^78^, ^81^, ^82^
C	CDH5	VE‐cadherin	1, 2, 3, 4	Blood	BBB derived and other organs	BBB derived	−	+/−	Indirect	−	Endothelial damage related but there are other CDH5 sources from the peripheral	^83^, ^84^, ^85^
C	CDH5	VE‐cadherin	1, 2, 3, 4	CSF	BBB derived and other organs	BBB derived	−	+	Indirect	+	Endothelial damage related and CSF compartment isolated from other sources of CDH5	^86^
C	CHI3L1 (YKL40)	Chitinase 3 like 1		Blood/CSF	Astrocytes, macrophages, chondrocytes, EC, vascular smooth muscle cells	Neuronal stress	−	−	Indirect	−	Upregulated in several neuronal and non‐neuronal pathologies	^78^, ^87^, ^88^
C	CLDN5	Claudin‐5	1, 2, 3	CSF	BBB and CP epithelial cells, other organs	BBB derived	−	+/−	Indirect	+/−	Functional protein at BBB and BCSFB	^54^, ^85^
C	CLDN5	Claudin‐5	1, 2, 3	Blood	BBB, CP epithelial cells, other organs	BBB derived	+	+/−	Indirect	−	Functional protein at BBB, BCSFB and other organs	^54^, ^85^
C	ENO2	Neuron‐specific enolase	3	Plasma/CSF	CNS and other organs	Neuronal damage and clearance	+/−	−	Indirect	−	Released after neuronal damage and is subsequently measured in CSF or plasma. No information regarding BCSFB available. Isotype differences require normalization of protocols.	^55^, ^77^, ^89^, ^90^, ^91^
C	GFAP	GFAP	3	Serum	Glial cells	Clearance	+	−	Indirect	−	Glial activation, released after neuronal damage and subsequently leaks over BBB. BCSFB data unavailable	^55^
C	HP	Haptoglobin/zonulin	1	Blood	Hepatocytes	BBB interfering	−	−	Indirect	−	Zonulin is able to interfere with TJs stability. Distinguishing haptoglobin and zonulin is challenging and often not specified	^49^, ^50^, ^71^, ^92^, ^93^, ^94^, ^95^, ^96^
C	ICAM	Intercellular adhesion molecule 1	1, 2, 3	CSF/plasma	BBB, CP, and other organs	BBB derived	+	+/−	Indirect	−	Neuro‐inflammation related, present at both the BBB and BCSFB.	^88^, ^97^, ^98^
R	N.A.	Immunoglobulins	3	CSF:serum	Immune system/glial cells	Leakage	+	−	Direct	−	Non‐specific for BBB since BCSFB passage possible; inconsistent results	^50^, ^77^, ^78^, ^80^
C	JAM1	Junction adhesion molecule 1	1	Blood	BBB and CP	BBB derived	+	−	Indirect	−	Expression is throughout the body	^54^
C	MAPT	tau	3	CSF	Neurons	Clearance	−	−	Indirect	−	Neuronal damage	^85^
C	MMP (protein group)	Matrix metalloproteinases	1	CSF and blood	BBB, CP, and other organs	BBB‐ECM degradation	−	+/−	Indirect	+/−	BBB ECM breakdown; both up‐ and downregulated in CSF	^77^, ^85^, ^99^, ^100^, ^101^, ^102^, ^103^
C	N.A.	Total protein	N.A.	CSF	CNS, BBB, BCSFB, blood	Leakage	+	−	Direct	−	Influenced by neuropathologies and BCSFB dysfunction. Various confounders described	^80^
C/R	OCLN	occludin	1, 2, 3	Blood and CSF	BBB and CP	BBB derived	+	+/−	Direct	+/−	Used as standalone BBB dysfunction biomarker in blood and CSF but a OCLN index has been used as well	^47^, ^54^, ^85^
C	PDGFR‐β	Platelet‐derived growth factor receptor‐beta	1, 2, 3, 4, 5	CSF	Pericytes and smooth muscle cells	NVU derived	+	+/−	Indirect	+/−	NVU‐related cells. Differences between pathologies and healthy controls are not well established	^34^, ^49^, ^50^, ^78^, ^104^
C	PECAM‐1	Platelet and EC adhesion molecule 1	1, 2, 3, 4	CSF/blood	Throughout vasculature	BBB derived	−	−	Indirect	+/−	BBB specificity in CSF > serum	^105^
C	s100b	S100 calcium‐binding protein B	5*	Serum	Astrocyte, Schwann cells, white and brown fat, skin, skeletal muscle	Clearance	+	−	Direct	+/−	Released after neuronal damage and subsequently leaks over BBB; uncertainties in flow exist	^55^, ^106^, ^107^, ^108^
C	TIMP4	Matrix metalloproteinase ‐11	1	CSF/blood	BBB and other organs	BBB derived	+/−	−	Indirect	+/−	BBB ECM breakdown; both up‐ and downregulated in CSF	^47^, ^48^, ^77^, ^85^, ^92^, ^109^
C	TJP1	Tight junction protein 1	1	CSF/blood	BBB, CP, other organs	BBB derived	+	+/−	Indirect	−	TJ expression is through‐out the body	^47^, ^54^, ^77^, ^85^, ^110^
C	VCAM1	Vascular cell adhesion molecule 1	1, 2, 3	CSF/blood	BBB, CP, other organs	BBB derived	+/−	+/−	Indirect	−	Endothelial inflammation marker, human CP expression uncertain	^88^, ^94^, ^98^

*Note*: −, low level; ±, uncertain level; +, high level; *, nonsignificant correlation.

Abbreviations: BBB, blood–brain barrier; BCSFB, blood–cerebrospinal fluid barrier; C, concentration in single fluid; CNS, central nervous system; CP, choroid plexus; CSF, cerebrospinal fluid; DCE‐MRI, dynamic contrast‐enhanced magnetic resonance imaging; ECM, extracellular matrix; GFAP, glial fibrillary acidic protein; N.A., not applicable; NVU, neurovascular unit; R, ratio between CSF and blood; TJ, tight junction.

#### 2.2.2 Fluid biomarkers and BBB biomarker selection criteria

No standalone fluid biomarker can establish a functional relationship with the BBB as it only reflects analyte concentration (criterion 1). For example, claudin‐5 is directly involved in EC integrity, whereas albumin is a blood‐derived protein and not directly structurally or functionally related to the BBB.^56^, ^57^ Mechanistic relevance should therefore be evaluated during analyte selection for targeted approaches or during analysis for untargeted approaches. This typically relies on complementary studies, including genomics and cell‐ or animal‐based experiments, to provide mechanistic insight into the relationship with the BBB.

Another major limitation of all fluid biomarker detection methods is the lack of spatial resolution, which compromises specificity (criterion 2). Because analytes are sampled from enclosed compartments, such as blood or CSF, their precise barrier or tissue of origin cannot be determined, and non‐BBB contributions cannot be excluded. BBB specificity must therefore be established from prior evidence or explicitly evaluated. Analyte origin can be assessed using complementary approaches, including *post‐mortem* immunohistochemistry (IHC), animal models, and single‐cell transcriptomics. Relevant source tissues depend on the sampled biofluid: for CSF, the BCSFB and neuronal cells must be considered, whereas for blood, peripheral vasculature and extracranial organs are potential contributors.^58^

Marker specificity varies substantially. PECAM1 is selectively expressed by BBB ECs and not by the BCSFB or other CNS cell types, making it relatively BBB‐specific in CSF, but not in serum due to widespread peripheral endothelial expression.^59^, ^60^ In contrast, TJ proteins, such as occludin and claudin‐5, are expressed at both the BBB and BCSFB, limiting BBB specificity when measured in CSF.^61^, ^62^, ^63^

When employing blood–CSF concentration ratios as indicators of BBB permeability, other brain barriers must be considered as potential entry routes, since the analyte could traverse over other (leaky) CNS barriers such as the BCSFB. This decreases their specificity for the BBB.^58^, ^64^, ^65^, ^66^ Accordingly, commonly used markers, such as the albumin quotient (Qalb), immunoglobulins (Igs), and total CSF protein, primarily indicate BCSFB rather than BBB permeability, with Qalb described as a specific BCSFB marker.^65^, ^67^, ^68^, ^69^, ^70^ Although BCSFB dysfunction is less extensively studied than BBB dysfunction, it has been reported in AD where reduced claudin‐5 expression in CP epithelium is associated with altered CSF albumin levels.^71^ Overall, because fluid biomarkers cannot resolve the route of barrier passage, they should be interpreted as measures of global blood–CNS barrier integrity rather than BBB‐specific permeability.

Fluid biomarker concentrations can be influenced by multiple confounders (criterion 3), and each biomarker may respond differently. For example, CSF albumin and total protein are affected by age and body mass index,^72^ whereas serum platelet‐derived growth factor receptor‐beta (PDGFR‐β) levels are associated with age and hypertension.^55^ In dementia studies, prior traumatic brain injury has been shown to potentially alter levels of CD147, transforming growth factor‐beta (TGF‐β), and S100 calcium‐binding protein B (S100B).^50^, ^73^, ^74^ Moreover, systemic inflammatory co‐pathologies can further influence BBB permeability via cytokine signalling.^75^ Common factors such as age and sex should therefore be considered, and additional confounders, including hypertension, kidney function, or relevant polymorphisms, should be incorporated when applicable. Technical confounders may also arise during the pre‐analytical phase, and adherence to established fluid biomarker protocols is strongly recommended to ensure harmonization.^76^

A major strength of fluid biomarkers is the ability to assess BBB dysfunction longitudinally in living patients (criterion 4). The duration of dysfunction, whether acute, subacute, or chronic, can affect biomarker levels, making careful timing of sample collection critical to avoid misinterpretation.^77^ Longitudinal monitoring can improve understanding of BBB dynamics and its role in neurodegeneration.

Independent validation of fluid biomarkers is essential (criterion 5). Correlation between different fluid biomarkers can provide insights into distinct BBB functions, and orthogonal validation can be achieved using in vivo imaging methods, such as dynamic contrast‐enhanced magnetic resonance imaging (DCE‐MRI) or arterial spin labeling (ASL). However, such studies remain rare, and few biomarkers consistently correlate across techniques. For instance, albumin ratios show inconsistent correlations with white matter permeability measured by DCE‐MRI,^78^, ^79^, ^80^, ^81^ and S100B does not correlate with DCE‐MRI after traumatic brain injury.^80^, ^82^

#### 2.2.3 Conclusion

Currently, no ideal fluid biomarker exists to measure BBB dysfunction, as each has strengths and limitations regarding the previously stated guidelines. Direct assessment of BBB dysfunction, that is, alterations in BBB permeability, can be achieved either by evaluating CSF‐to‐blood ratios or indirectly via expression of specific BBB‐associated biomarkers in CSF. However, for ratio‐based approaches, the permeability of the BCSFB remains insufficiently characterized for most proteins, but should be defined to ensure specificity for the BBB. Alternatively, the use of indirect but BBB‐specific targets can indicate disturbances of the BBB or the NVU in neurodegenerative diseases. Both established and novel BBB biomarkers should be critically (re‐)evaluated and validated with independent data and non‐fluid BBB measures to improve the accuracy and reliability of BBB dysfunction assessments.

## 3 IN VITRO MODELS OF BBB DYSFUNCTION

In vitro models offer valuable insights into the underlying pathological processes related to BBB changes by enabling controlled studies of cellular interactions. They allow researchers to isolate specific aspects of the complex interplay between human‐specific BBB cell types, providing a clearer understanding of the mechanisms that contribute to BBB function and pathology. The possibility to introduce external factors into cell culture medium or altering genes within specific cells makes in vitro models a useful tool to assess BBB function directly as well as indirectly.

### 3.1 Brain ECs

Since brain ECs are the central player of the BBB, there is a special focus on these cells in the in vitro studies of BBB functioning. Different types of ECs are being used, such as human primary cerebral ECs, which constitute an excellent model due to their genetic and epigenetic relevance to human brain vasculature. However, obtaining primary human brain ECs and keeping them in culture comes with a number of practical challenges, due to the changing phenotype over time and the difficulty of obtaining the right samples. Alternatively, immortalized human brain EC lines, such as hCMEC/D3, are more practical as they can be cultured easily.^83^ They represent key elements of ECs and are widely used, but in terms of the pathological characteristics, immortalized cells lack genetic, epigenetic, and physiological features.^84^ One approach to addressing this is the use of human induced pluripotent stem cells (hiPSCs).^85^ These hiPSCs can be differentiated into ECs with human brain‐specific features. However, the current differentiation protocols are limited and often exhibit epithelial‐like features. These cells show a high transcellular resistance and express key transporters of the brain vasculature, such as P‐gp, which makes them a suitable model for drug transport research.^86^

The expression of these transporters, including P‐gp, breast cancer‐resistant protein (BCRP), and glucose transporter‐1 (GLUT1), is a crucial but indirect proxy for BBB functioning and has been related to various pathological settings.^87^ The expression levels of these proteins in cells can be microscopically visualized using immunocytochemistry (ICC), which is an antibody‐based staining technique. The same accounts for the expression of junction proteins, most prominently VE‐cadherin, claudin‐5, occludin, and ZO‐2, which line the cellular membrane of ECs in an in vitro monolayer.^88^ Additionally, the endothelial response to inflammatory stimuli, for example, the tumor necrosis factor alpha (TNF‐α), which has been shown to upregulate cellular adhesion molecules (CAMs), provides a proxy of endothelial barrier function. Key inflammatory response molecules include E‐ and P‐selectin, ICAM1, and VCAM1, which as binding partners of immune cells moderate their trafficking through the BBB.^89^ Despite the ease and value of these assays, to directly test the functionality of the BBB, the use of more complex models is inevitable.

### 3.2 Complex in vitro models

A variety of complex in vitro models aim to represent the functionality of the BBB. The transwell system, for example, is a widely used model for investigating BBB dysfunction directly via a paracellular permeability assay. ECs are cultured on a coated porous membrane, forming a tightly connected monolayer with TJs and AJs. This mimics the barrier function of the BBB and allows for a number of assays, often using fluorescent dextran tracers to measure permeability through the monolayer. To add more complexity, pericytes can be cultured at the other side of the membrane and astrocytes or other secreting cells at the bottom of the well. This way, the transwell system can be used to measure permeability under the influence of coculture with other cell types or the addition of inflammatory markers. Another validation method to directly assess barrier function is via trans‐endothelial electrical resistance (TEER) measurement, which uses an electrode to measure the tightness of the trans‐endothelial connectivity.^90^ Again, the addition of certain inflammatory markers resulted in the reduction of the TEER, providing another functional readout of BBB permeability.^91^ A major advantage of the transwell system is that after the completion of the functional experiments, the cells can be measured for altered expression of junction proteins, allowing for making a connection between functional and structural changes. A common way to measure TEER is by means of electric cell‐substrate impedance sensing (ECIS) which provides real‐time data on TEER on a monoculture of ECs.^92^ More recently, this real‐time device is available for the transwell system as well, making it possible to relate TEER results to cocultures, ICC, or a dextran permeability assay.^93^ These assays are therefore highly practical and informative but neglect the complexity of certain cellular interactions, ECM, shear stress, and flow.

Coculture of ECs with pericytes and astrocytes already enhances the physiological relevance by producing cell matrices and intercellular receptor–ligand interactions. The use of cerebral organoids, derived from hiPSCs, allows for further advance modeling of these interactions through a 3D coculture, embedded in a nutrient‐rich gel. However, cerebral organoids are unsupervised structures, which makes it difficult to successfully vascularize these models and deal with the large structural variability.^94^ Organ‐on‐chip technologies provide an answer to this problem through dynamic platforms hosting microfluidic channels, porous membranes, and hydrogels, tightly controlled but connected.^95^ Designs range from membrane‐based “sandwich” models to micropillar‐separated chambers and sacrificial template‐based channels.^96^, ^97^ Current efforts in the microfluidics field are to provide a microfluidics platform with a vascular bed that allows for perfusion of the attached organoid. The microfluidics models that are on the market already facilitate the formation of complex vascular networks with pericyte coverage and a perfusable lumen that is connected to a pump to induce flow. Studying permeability (e.g., dextran leakage) in such a system provides a more physiologically relevant alternative to a simple monolayer. The field is moving fast, with many new technologies and protocols being published but these models come with challenges including high cost, reproducibility, and standardization.

### 3.3 Discovering BBB‐dysfunction biomarkers with in vitro models

Each cellular model presents its unique strengths and limitations, making the research question important for the selection of the appropriate technology. Transwell systems and microfluidics provide a powerful tool to study cellular interactions and permeability, thereby demonstrating mechanistic and functional relevance. Recent 3D models have significantly improved this by mimicking the physiological architecture of the BBB. These setups allow for linking the barrier function to the RNA and protein levels of markers in specific BBB cell types (criterion 1). Two‐dimensional cultures and separation of individual cell types offer a detailed analysis, enabling a high degree of specificity. For example, when isolating endothelial monolayers, biomarkers can be linked to the barrier interface without interference from glial or neuronal cells (criterion 2). Advances in hiPSCs have improved specificity and translatability of cellular models; however, they have also introduced potential confounders through technical variations in differentiation protocols, donor variability, and cell maturation, which can introduce significant experimental bias unrelated to the pathology (criterion 3). These limitations can be controlled by using a sufficiently balanced and sized cohort of donors (biological replicates) and repeat experiments with multiple differentiations (technical replicates). An immense benefit of in vitro models is their temporal resolution, as they allow for the precise manipulation and measurement of functional or structural changes. Through modern measurements of electrical resistance or leakage, permeability assays yield information on responses to stimuli in real time (criterion 4). Finally, regarding independent validation, the choice of model heavily impacts reproducibility (criterion 5). The cost‐efficiency of 2D systems facilitate replication across laboratories, while the increased variability and complexity of 3D models remain a challenge for standardization and routine validation.

## 4 *POST‐MORTEM* BBB DYSFUNCTION METHODS

*Post‐mortem* studies provide high‐resolution direct or indirect insights into BBB dysfunction by examining animal or human tissues with a preserved native cellular architecture. Common methods include IHC and immunofluorescence (IF), transmission electron microscopy (TEM), and tracer‐based methods. These methods rely on widely available reagents and can use biobank samples, increasing accessibility and applicability. However, tissue quality can vary substantially, particularly in human samples. Animal tissue processing can be tightly controlled and optimized, which enhances tissue quality and enables the use of exogenous tracers in vivo to assess *post‐mortem* BBB leakage. In contrast, human samples often differ in *post‐mortem* intervals, preservation techniques, and storage conditions, all of which can affect tissue integrity and staining efficacy.^98^, ^99^, ^100^

### 4.1 IHC and IF

IHC and IF are antibody‐based techniques for detecting specific proteins in tissue whereas ICC is typically applied to isolated cells or cell cultures. IHC relies on enzymatic reactions from chromogen‐conjugated antibodies visualized by light microscopy, while IF uses fluorophore‐conjugated antibodies and fluorescence microscopy. Both are semi‐quantitative, allowing fluorescence intensity or signal structure quantification. However, quantifying intensity requires consistent protocols, timing, and imaging parameters for reliable comparisons.

**FIGURE 3:**
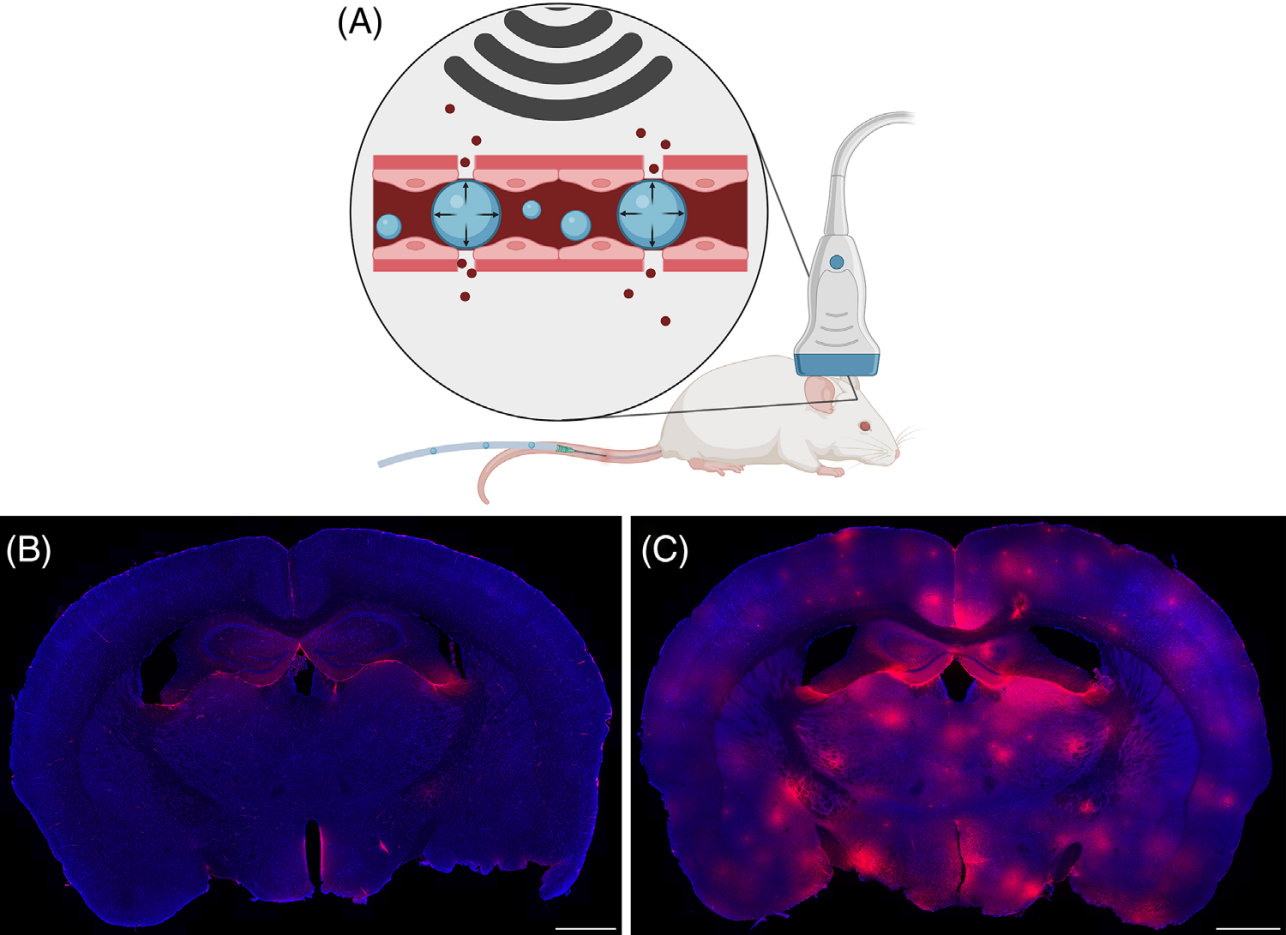
Typical example of immunofluorescence (IF) microscopy images following blood–brain barrier (BBB) opening in mice. (A) Schematic overview of BBB opening experiment using sonopermeation. Healthy anesthetized adult mice are subjected to high‐power diagnostic ultrasound combined with intravascular infusion of microbubbles. The microbubbles will oscillate due to ultrasound exposure, causing a mechanical force on the endothelial cells that opens the tight junctions. (C) Coronal mouse brain sections stained for Ig show widespread leakage after the BBB opening procedure whereas control mice that received only ultrasound without microbubbles show minimal Ig presence, which remains restricted to specific brain regions (B). Cell nuclei are stained with DAPI (blue), and endogenous Ig (red) indicates BBB leakage. Scale bars = 1 mm. Unpublished results from van der Panne et al.

In BBB research, IHC/IF indirectly assesses dysfunction by examining the structural localization of NVU proteins, including cell‐specific markers as well as TJ, AJ, BM, and transporter proteins. IHC/IF also directly assess dysfunction by detecting serum proteins (e.g., albumin, Ig) in CNS, serving as endogenous BBB leakage markers (Figure 3) which eliminates the need for tracers and enabling use in human tissue.^101^ Interpretation must consider that some serum proteins (e.g., haptoglobin, transferrin) are endogenously expressed in the brain parenchyma,^102^, ^103^ while others (e.g., albumin, Ig) are normally found in CSF and certain brain regions (Figure 3B).^104^, ^105^, ^106^ Their presence alone does not confirm BBB dysfunction as elevated levels may reflect BCSFB leakage or protein upregulation. Localization of these serum proteins (e.g., near periventricular spaces, around the microvasculature) using IHC and IF helps differentiate BCSFB from BBB leakage, thereby offering greater spatial specificity than targeting serum proteins as a fluid biomarker. In animals, exogenous tracers offer a more reliable alternative to control for endogenous expression.

IHC and IF enable multitarget detection with high specificity. However, marker selection is crucial as some proteins (e.g., α‐smooth muscle actin) are expressed in multiple cell types, while soluble proteins may be lost during tissue processing steps.^101^, ^107^ Multiplexing and the technique's high spatial resolution can offset reduced marker specificity by providing spatial context. Furthermore, practical factors, including protocol design and antibody quality, can impact results by influencing signal intensity, binding affinity, non‐specific binding, and antigen masking.^107^, ^108^

### 4.2 *Post‐mortem* assessment of exogenous tracer leakage

A useful way to directly study BBB dysfunction in animal models is by utilizing intravascular (IV)‐injected exogenous tracers (dyes or fluorescent molecules) to assess permeability. These tracers normally do not cross the BBB due to their molecular characteristics (size, lipophilicity, charge).^109^ When BBB integrity is compromised, they leak into the parenchyma via transcellular and/or paracellular routes, allowing visualization and quantification of leakage. Tracers are versatile as they can be employed to study BBB leakage, both in vivo using intravital microscopy and *post‐mortem* following *ante mortem* injection. After death, these tracers provide valuable insights into BBB dysfunction at the time of death, but careful consideration of experimental timing is essential. The interval between tracer injection, death, and fixation can affect tracer distribution in the tissue and therefore data interpretation. Due to the need for precise timing and possible toxicity, tracer use is restricted to animals.

Commonly used tracers to assess BBB dysfunction include Evans blue (EB) and fluorescently labeled dextrans, but there are many more.^110^ These tracers allow leakage visualization and semi‐quantification using microscopy and can be quantitatively measured using spectrophotometry or spectrofluorometry.^109^, ^110^, ^111^, ^112^ EB (961 Da) is a widely used dye that binds to albumin (69 kDa when bound) and additionally allows for macroscopic leakage detection.^109^, ^110^, ^112^ It is cost‐effective and easy to use but toxic in vivo and limited to detecting leakage for molecular sizes ≥69 kDa. EB quantification has limitations: Free EB may cross the BBB more easily, solvents alter EB structure, and non‐specific albumin binding complicates interpretation.^110^ Fluorescently labeled dextrans are non‐toxic and available in various molecular weights (3 to 2000 kDa), allowing detection of minor and major leakages.^110^, ^113^ While more versatile and reliable than EB, they are more expensive and not visible macroscopically, only microscopically. The choice of tracer depends on experimental goals: EB is suitable for rapid, large‐scale screening of significant BBB leakage, dextrans for detailed, size‐dependent permeability assessment.

### 4.3 Transmission electron microscopy

While IHC and IF provide high‐resolution imaging of BBB structure and dysfunction, TEM offers even greater detail. TEM is a super‐high‐resolution imaging technique that employs electron beams to visualize cellular and subcellular structures. It can indirectly assess BBB dysfunction via visualization of structural NVU and BBB components, including ECs, pericytes, astrocytes, basal lamina, TJs, and EC vesicles.^114^, ^115^, ^116^ Though primarily qualitative, it can be employed to quantify TJ and vesicle numbers,^114^, ^116^ although sufficient tissue quality and contrast is required, which is heavily dependent on the sample preparation, post‐staining steps, and imaging settings.^117^ IHC, IF, or immuno‐EM may be preferred for the assessment of TJs and vesicles due to improved signal‐to‐noise ratio (SNR) through specific marker targeting. The primary limitations of all TEM techniques are its time‐consuming nature and technical complexity, making it challenging to apply to large tissue samples and thereby increasing the risk of sampling bias.

Immuno‐EM combines TEM resolution with IHC specificity using electron‐dense particles, like gold, conjugated to antibodies to target and localize BBB‐related structural proteins (e.g., TJs, transporters) with high precision as an indirect measure of BBB dysfunction.^118^, ^119^, ^120^ Despite exceptional resolution and specificity, it is costly, technically demanding, and, like IHC, highly dependent on antibody and protocol quality.

With IV‐administered electron‐dense tracers (e.g., colloidal gold, horse radish peroxidase, lanthanum), TEM enables direct assessment of BBB dysfunction via the precise localization of tracer leakage (e.g., in EC vesicles or TJs).^114^, ^116^, ^119^ Tracer choice is critical due to distinct permeability characteristics which influences results and data interpretation. Because assessment is limited to small samples, detecting vessels with leakage, especially subtle or focal leakage, can be challenging. TEM tracers are costly and limited to animal models, yet show the most superior spatial resolution for visualizing BBB dysfunction.

### 4.4 Discovering BBB‐dysfunction biomarkers with *post‐mortem* methods

IHC, IF, and immuno‐EM can assess BBB dysfunction indirectly via targeting of BBB‐associated structural proteins, such as TJ or transporter proteins, or directly via targeting of blood‐derived proteins as permeability indicators. Marker specificity and mechanistic relevance vary widely, making careful selection essential (criteria 1 and 2). Some markers have a clear mechanistic link, while others may reflect non‐BBB or multiple different BBB sources, like PDGFR‐β, which is not only expressed by pericytes, but also by astrocytes and fibroblasts.^101^ Exogenous tracers provide a functional readout of BBB permeability in animals (criterion 1). Tracer specificity, including potential leakage pathways, depends on established tracer characteristics (criterion 2).

*Post‐mortem* approaches lack temporal resolution, reflecting BBB structure only at death (criterion 4). Exogenous tracer leakage reflects BBB permeability at the interval between administration and death, while endogenous tracers, such as blood proteins, reflect permeability shortly before death. Acute, semi‐acute, or chronic BBB dysfunction may produce different marker expressions or permeability levels and should be considered. Interpretation of tracer leakage is further influenced by ongoing tracer clearance (criterion 3). Other potential confounders, including neurovascular comorbidities or age, should be addressed through exclusion or appropriately matched controls. The results of *post‐mortem* approaches are generally semi‐quantitative, and proteomics or single‐cell transcriptomics can provide quantitative validation (criterion 5). Low temporal resolution and limited quantification possibilities can be mitigated by validating tracer‐based findings with in vivo permeability methods such as DCE‐MRI or ASL.

## 5 IN VIVO IMAGING OF BBB DYSFUNCTION

While *post‐mortem* techniques provide valuable high spatial resolution insights, they lack temporal resolution and cannot capture the dynamic nature of BBB dysfunction. In contrast, in vivo imaging enables real‐time assessment of BBB dysfunction in both animal models and humans, making it well suited for longitudinal studies on disease progression and interventions. Owing to their non‐invasive nature, MRI and positron emission tomography (PET) are particularly applicable in clinical settings. These modalities hold potential for early detection of BBB impairment, monitoring of disease evolution, and guiding timely interventions to mitigate further damage. Contrast‐enhanced and water‐exchange MRI, but also specific PET tracers or fluorescent exogenous tracers in combination with intravital microscopy, can be utilized to directly measure BBB dysfunction by assessing BBB permeability. Targeting NVU cells or BBB‐related proteins using intravital microscopy or PET tracers provides an indirect measure of BBB dysfunction.

### 5.1 Intravital microscopy

Intravital microscopy uses any type of fluorescent microscopy to study biological processes, such as BBB dynamics, in real time with high spatial and temporal resolution in vivo. This approach allows researchers to study BBB integrity in vivo, observing complex interactions between cell types and various biological systems in their natural context. This complex interaction cannot be captured with in vitro models due to the lack of systemic influences such as immune responses, blood pressure, heart rate, and respiratory rate. By enabling continuous live imaging of the cerebrovasculature, intravital microscopy provides critical insights into BBB structure and permeability across development, pathology, and interventions.^121^, ^122^ It can be used to directly or indirectly assess BBB dysfunction by assessing permeability using fluorescent tracers^113^, or by labeling BBB‐associated molecular and cellular targets via genetic manipulation,^123^ intravascularly injected fluorescent antibodies,^124^ or lectins^125^ respecively. Experimental factors like anesthesia, oxygenation, and brain temperature must be carefully controlled as these can impact BBB integrity in vivo.^126^, ^127^, ^128^ Awake multiphoton imaging is increasingly used in rodents to eliminate anesthesia effects, enabling observation of BBB dynamics during both sleep and wake states.^129^ These methods are currently restricted to animal models due to size restrictions and limited optical access to living human brain tissue.

#### 5.1.1 Fluorescence imaging

In vivo fluorescence imaging using confocal or light‐sheet microscopy provides real‐time, high‐resolution assessment of BBB dysfunction.^118^, ^130^, ^131^ However, these techniques are limited to small, transparent organisms due to light penetration constraints.^132^ Zebrafish larvae are the preferred model because they are easily genetically modified and their BBB closely resembles that of mammals.^133^, ^134^ Their small size enables whole‐brain fluorescence imaging, unlike rodent multiphoton imaging, which is restricted to smaller brain regions. Although zebrafish larvae are mostly transparent, various techniques can further enhance their optical clarity and spatial resolution.^135^, ^136^ Confocal microscopy is widely used for detailed 4D imaging but can cause phototoxicity and photobleaching, especially during long‐term imaging. Light‐sheet microscopy enables faster imaging with reduced phototoxicity and photobleaching by illuminating only a thin slice of the sample at a time, making it well suited for extended imaging sessions.^132^, ^137^

Zebrafish larvae have key advantages, including easy care, high egg yield, and rapid development.^138^ Despite these advantages, zebrafish models have limitations. They must be anesthetized and immobilized during imaging, which may influence BBB function.^139^ While the zebrafish BBB shares many key features with the mammalian BBB, differences remain, including variations in astrocyte types, pericyte origins, and endothelial transporters.^138^ Additionally, zebrafish lack the full complexity of the mammalian neuronal and cerebrovascular networks. Despite these challenges, zebrafish remain a cost‐effective, accessible model that provides invaluable real‐time, high‐resolution insights into whole‐brain BBB dysfunction development and maturation, which is impossible to achieve in mammals.

#### 5.1.2 Multiphoton imaging

Multiphoton imaging excites tissue by the simultaneous absorption of two or more longer wavelength photons.^140^ This enables tissue imaging with more than twice the depth and less phototoxicity as compared to confocal microscopy, even reaching a depth of 900 µm.^140^, ^141^ This makes it more ideal for in vivo studies in rodents, which exhibit a more complex vascular and neuronal network compared to zebrafish, thereby enhancing translatability to humans. Accessing the rodent brain for imaging usually includes procedures such as skull thinning or invasive cranial window placements. The thinned‐skull method and chronic cranial window enables longitudinal studies of pathology progression or recovery over a period of several weeks to months.^142^, ^143^

Despite its advantages, multiphoton imaging has limitations. Thinned‐skulls and especially cranial windows are technically challenging and can induce chronic inflammation, scar tissue formation, and bone regrowth, which may obscure the imaging region. In addition, the drilling process can cause bleeding and acute inflammation, potentially leading to inaccurate interpretations of BBB leakage when imaging is performed immediately after surgery.^141^ To mitigate these effects, the use of chronic cranial windows or thinned‐skull preparations is recommended, and new techniques are being developed including skull optical clearing, which enhances spatial resolution without requiring skull removal.^144^, ^145^ Additionally, the imaging window is limited to the most superficial cerebral vessels and to a small area ranging from 0.2 to 5 mm in diameter, thereby making the choice of window placement crucial for accurate data interpretation.^141^ Furthermore, a multiphoton microscope is more expensive and less accessible compared to a standard fluorescence microscope. However, this method remains invaluable for capturing high‐resolution spatial and temporal insights into BBB dysfunction and structure in a more complex biological system.

#### 5.1.3 Discovering BBB‐dysfunction biomarkers with intravital microscopy

Intravital imaging enables indirect in vivo assessment of BBB dysfunction by fluorescently labeling BBB‐associated molecular and cellular targets. As with *post‐mortem* markers, the specificity and mechanistic relevance to the BBB is heavily dependent on the chosen target (criteria 1 and 2). Combined with fluorescent tracers, intravital microscopy directly measures BBB dysfunction by assessing permeability, with specificity (e.g., leakage pathway) determined by tracer properties (criteria 1 and 2). Experimental confounders, such as blood flow and respiratory rate, should be carefully controlled, while factors like age and sex should be addressed using appropriate controls (criterion 3). The high temporal and spatial resolution of intravital microscopy, together with longitudinal imaging, enables evaluation of acute, semi‐acute, and chronic BBB dysfunction and their progression or recovery over time (criterion 4). Like *post‐mortem* imaging techniques, intravital microscopy is largely semi‐quantitative, and reliability is enhanced by validating target expression with proteomics or single‐cell transcriptomics and tracer‐based permeability with DCE‐MRI or ASL (criterion 5).

### 5.2 Contrast agent‐based MRI

In research on neurodegenerative and neurovascular diseases, in vivo imaging of patients is crucial for understanding human BBB (dys)function and for future translation of preclinical findings into clinical interpretations and applications. MRI is a key imaging modality enabling this translation. It relies on the magnetic properties of tissue water, in particular the energy absorption of electromagnetic waves and magnetic states of water molecules, and measuring their subsequent relaxation in a strong magnetic field. These relaxation properties differ per tissue type and can be influenced by exogenous paramagnetic substances such as gadolinium‐based contrast agents (GBCA).^146^ These are relatively large molecules (typically 547 to 668 Da),^147^ which are generally restricted from entering brain tissue by the TJs of the BBB.^148^, ^149^ Accumulation of these GBCA in brain tissue is therefore generally interpreted as a direct measurement of passive paracellular BBB leakage through TJ disruptions. Clinically, post‐contrast MRI scans are a critical step in the evaluation of cancer in the brain, as neovasculature associated with gliomas lacks a proper BBB and therefore shows extravascular contrast agent accumulation.^150^ In patients with neurodegenerative disease, much more subtle and diffuse BBB leakage is expected.^151^ Therefore, quantitative approaches to measure BBB leakage are more suitable than visual assessment in this population.^152^ Two techniques exist that directly measure BBB dysfunction by quantification of BBB permeability to GBCA. However, one of these, dynamic susceptibility contrast (DSC) MRI, which relies on the disturbance of the highly homogeneous magnetic field, has much lower ability to discriminate low contrast agent concentrations in tissue from intravascular contrast agent and is therefore not recommended to measure subtle BBB leakage.^153^ The other method, DCE‐MRI, is the most commonly used technique to measure (subtle) BBB leakage in humans and animals.^153^

#### 5.2.1 DCE‐MRI

**FIGURE 4:**
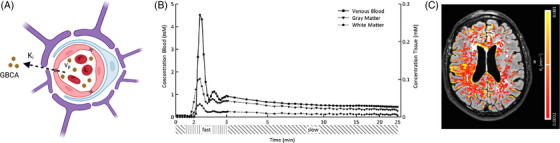
Overview of dynamic contrast‐enhanced (DCE)‐MRI‐based assessment of BBB leakage. (A) Intra‐ and extravascular gadolinium‐based contrast agent (GBCA) signal can be used to quantify the leakage rate (*K_i_*) and the blood plasma fraction (*v_p_*). (B) Dynamic concentration curves of GBCA distributed over blood and brain tissue and (C) resulting leakage rate (*K_i_*) map in patient with early Alzheimer's disease. Adapted from van de Haar et al.^36^ with permission.

DCE‐MRI builds upon the previously described principles, acquiring a dynamic sequence of T1‐weighted images before, during, and after GBCA administration. The GBCA increases the T1 relaxation rate in proportion to its concentration, allowing for the quantification of contrast agent concentration in tissue.^146^, ^154^ In DCE‐MRI, leakage through the BBB is quantified by the leakage rate (*K_i_*), which equals the product of the permeability and the vascular surface area (*K_i_* = PxS) for low leakage rates (so‐called permeability limited regime, where permeability‐surface [PS] area << blood flow). The BBB leakage rate can be calculated by fitting the GBCA concentration curves of the tissue and the blood (the vascular input function) to a pharmacokinetic mathematical model (Figure 4B). While many such models have been used, the Patlak method^155^ has proven most accurate and least complex in low‐leakage regimes, as seen in patients with neurodegenerative diseases (*K_i_* < 0.015 min^−1^). This method quantifies GBCA leakage from the vessels to the extravascular extracellular space and the blood plasma fraction (*v_p_*) (Figure 4A). This method assumes that back‐diffusion of GBCA to the intravascular space is negligible, a valid assumption in low‐leakage regimes and with normal measurement times (<30 min).^156^, ^157^ While global consensus recommendations for DCE‐MRI acquisition exist to maintain consistent standards and cross‐study comparability,^158^ ongoing research continues to refine the technology, thereby improving spatial/temporal resolution, SNR, and time efficiency.^159^

Future improvements in modeling and data analysis have the potential to enhance DCE‐MRI's capabilities to detect subtle leakage variations and patterns, particularly now that high‐resolution protocols are available.^159^, ^160^ For instance, physics‐informed neural networks^161^ and Bayesian fitting methods^162^ have shown the potential to improve parameter fits for noisy data. Additionally, relating DCE‐MRI‐based BBB leakage to CBF,^163^ water transport across the BBB,^164^ or vessel architecture imaging^165^ can yield new insights into cerebrovascular pathology.

#### 5.2.2 Contrast agent‐based probing of transporter function

While the disruption of TJs, connecting ECs, is thought to be the prime focus of BBB imaging, expanding contrast‐enhanced approaches to active transport mechanisms will require the adoption of novel contrast agents.^146^ The most promising contrast agent in this regard is D‐glucose, an endogenous biodegradable metabolite.^43^ This contrast agent passes the BBB mainly via the GLUT1 transporter, whose reduced expression has been associated with AD and cognitive impairment.^166^, ^167^ D‐glucose concentration can be quantified using glucose chemical exchange saturation transfer (glucoCEST). This technique offers insights into glucose transport across the BBB and its subsequent metabolism and provides another way to directly assess BBB dysfunction using MRI by assessing permeability via this transcellular pathway.^168^, ^169^ However, tissue‐specific metabolic demand must be considered for the interpretation of these concentrations, and this technique requires advanced MRI hardware (field strength, selectivity of the radiofrequency pulse).^43^ While promising, such transporter‐targeted contrast approaches require further technical maturation and validation before they can be implemented as robust BBB (dys)function biomarkers in clinical cohorts.

#### 5.2.3 Discovering BBB‐dysfunction biomarkers with DCE‐MRI

DCE‐MRI fulfills criteria 1 and 2, as it enables a direct in vivo assessment of BBB leakage through disrupted TJs in humans and animals. Measuring this physical barrier function complements the contrast agent probing transporter function, because paracellular leakage could reduce their specificity by introducing an additional route across the BBB.

Despite the increasing use of DCE‐MRI to quantify BBB leakage in patients with neurodegenerative and/or neurovascular disease, DCE‐derived leakage metrics are sensitive to methodological variability and confounders (criterion 3), which can influence the measured leakage rate (*K_i_*).^170^ Quantitative BBB leakage outcomes can be very heterogeneous and suffer from a low SNR of the leakage measurements. For instance, some studies found stronger BBB leakage with increased cerebral small vessel disease severity,^171^, ^172^ whereas other studies found no such relationship.^173^, ^174^ Comparisons between studies or meta‐analyses are challenging due to methodological variations in acquisition and post‐processing, which sometimes leads to order‐of‐magnitude differences between reported BBB leakage rates.^158^ While methodological differences can be justified for innovative approaches, such differences hamper direct comparisons between studies from different research sites. Comparison studies would require participants to receive multiple doses of GBCAs, each separated by enough time to ensure complete excretion of the previously administered GBCA, which poses practical limitations.

Since *K_i_* is the product of the vascular permeability and the vascular surface area, decreases in the surface area due to microvascular rarefaction would cause a decrease in *K_i_*.^149^ Similarly, local decreases in microvascular blood flow can also reduce the measured *K_i_*, as less GBCA is supplied to the region.^170^ Both alterations in blood flow and vascular density lower the ability to detect BBB dysfunction. Additionally, the measured BBB leakage indicates specifically GBCA leakage and it is therefore challenging to extrapolate this measure to passive leakage of lower‐molecular‐weight substances or to active transport of biologically relevant substances like proteins.^158^

Regarding temporal considerations (criterion 4), DCE‐MRI typically quantifies permeability over a 15‐ to 30‐min acquisition window and is therefore unable to capture changes in BBB permeability on a shorter timescale. For repeat acquisitions in patients with normal renal function, the recommended waiting time between successive doses is 12 h, leading to near complete clearance.^175^

Independent validation (criterion 5) for DCE‐MRI is complicated due to its specificity to paracellular leakage. Consequently, techniques probing para‐ and transcellular pathways (e.g., ASL‐based water‐exchange methods) may show positive, negative, or absent associations with DCE‐MRI, depending on region and disease.^176^, ^177^ As mentioned earlier, fluid biomarkers show similarly inconsistent relations to DCE‐MRI. In animal models, DCE‐MRI‐based permeability has been validated against dye/tracer extravasation.^178^, ^179^ In humans, validation between techniques focusing on paracellular leakage is limited. However, DCE‐MRI‐based leakage was found to be related to 99mTc‐DTPA uptake using single‐photon emission computerized tomography (SPECT), further supporting a paracellular leakage pathway.^82^, ^180^

### 5.3 Water‐exchange MRI

Contrast‐based MRI methods could be considered less effective at detecting very subtle BBB permeability changes, as GBCA molecules are still relatively large and leak slowly across the BBB unless TJs are severely disrupted.^148^, ^181^, ^182^ To address this limitation, newer MRI techniques instead quantify water exchange across the BBB without the use of contrast.^164^

#### 5.3.1 Multi‐echo time (multi‐TE) and diffusion prepared (DP) ASL

ASL non‐invasively labels water molecules in blood flowing in feeding arteries in the neck by magnetically inverting their spins, allowing them to be tracked as they perfuse the brain and extravasate across the BBB. Both multi‐TE^183^, ^184^, ^185^, ^186^, ^187^ and DP ASL^188^, ^189^, ^190^ quantify water exchange by distinguishing MRI signals arising from labeled water molecules within capillaries from those that have crossed the BBB into extravascular tissue. This is achieved by exploiting the fact that these two compartments have considerably different T2 relaxation times (multi‐TE) or diffusion properties (DP ASL). Data are fitted to dynamic models that estimate a rate of water transport, *k_w_*, between the compartments.

Like all ASL techniques, these sequences suffer from low SNR due to the label signal representing only 1% to 2% of the total tissue signal, requiring long scan times and introducing instability in *k_w_* estimation. For multi‐TE ASL, the models frequently assume constant blood T_2_ values, which could impact *k_w_* quantification. For DP ASL, the model relies on perfectly nulling all vascular signals with diffusion gradients, which may not always be achieved. Multi‐TE ASL studies report increased water exchange with age,^186^, ^191^, ^192^ while DP ASL studies show a decrease.^191^, ^193^ These contrasting results raise questions about whether both methods are truly measuring the same underlying exchange dynamic processes. Both methods rely on estimating arterial transit time (ATT) to quantify exchange rates. Morgan et al.^191^ reported significant differences in ATT estimates between the two approaches, which likely affected the calculated exchange rates. Future studies should carefully consider the impact of misestimated ATT on results, particularly in the context of neurodegeneration and aging that may result in regional ATT changes.^194^

#### 5.3.2 Water‐extraction‐with‐phase‐contrast‐arterial‐spin‐tagging (WEPCAST)

The ASL aforementioned methods measure signals when spins are in capillaries or have perfused into gray matter. In contrast, WEPCAST technique measures further into the vascular tree, capturing ASL signals from spins that have reached venous vessels.^195^, ^196^, ^197^, ^198^ Measurements are made in large draining veins, such as the superior sagittal sinus (SSS), where the net signal has contributions from spins that bypassed tissue exchange, making it possible to estimate the rate of water exchange across the BBB.

WEPCAST, by distinguishing compartments through flow velocity and spatial location at the end of the vasculature in the SSS, may offer a more reliable separation of spins that have and have not crossed the BBB compared to multi‐TE and DP ASL. However, as a consequence, WEPCAST provides only a global measure and not spatial maps, making it less effective for detecting region‐specific BBB pathology. Furthermore, errors are introduced from region‐of‐interest delineation in the SSS and low venous signal, since ∼95% of labeled spins extravasate across the BBB.^197^

#### 5.3.3 Filtered‐exchange imaging (FEXI)

Like DP ASL, FEXI leverages differences in diffusion properties between tissue compartments to measure water exchange.^199^, ^200^, ^201^ However, instead of labeling water via inversion of spins in brain‐feeding vessels, it selectively suppresses signal from blood flow in the microvasculature, thereby reducing measured diffusivity. The rate at which diffusivity returns to equilibrium, known as the apparent exchange rate (AXR), reflects water‐exchange dynamics between the two compartments.

One advantage of FEXI compared to the ASL methods is that it does not require any pre‐exchange transit times to be estimated.^202^ However, the AXR parameter is hard to interpret and requires separate blood volume measurements for conversion into exchange rates comparable to those obtained from ASL. The AXR model also does not account for intercompartmental differences in relaxation times. Powell et al.^203^ addressed these shortcomings by explicitly modeling compartmental exchange between blood and tissue. However, fixing blood volume fractions in this model could introduce significant errors, particularly if pathology‐related blood volume variations are expected.

#### 5.3.4 Discovering BBB‐dysfunction biomarkers with water‐exchange MRI

Water‐exchange methods provide a direct measure of a specific BBB function, namely, permeability to water (criterion 2). However, whether this implies that it therefore fulfills criterion 1 can be argued, since the broader functional relevance of this biomarker for assessing disease‐related BBB impairment remains uncertain. A potential role in brain clearance is hypothesized, and it might be correlated with the permeability of larger solutes such as proteins or toxins. Unlike GBCAs, which follow paracellular routes, water crosses the BBB via multiple pathways: transmembrane, TJs, and aquaporin‐4 (AQP4). This complicates interpretation (criterion 2) as changes in measured water exchange can arise from diverse mechanisms, and current imaging techniques cannot resolve these routes at the cellular level.^176^, ^204^, ^205^ Different pathway dysfunctions that could be co‐occurring in neurodegenerative diseases may even produce opposing effects, for example, reduced AQP4‐mediated uptake versus increased paracellular leakage. Moreover, a key unresolved issue is the actual timescales on which water exchanges between compartments and thus the processes that water‐transport MRI is sensitive to.

In addition, water transport changes may be influenced by confounders (criterion 3), for example, reflecting edema, rather than indicating pathological breakdown, and ASL‐based measurements could be influenced by hemodynamic changes. For multi‐TE and DP ASL, additional confounds arise from pathological or demographic factors that alter CBF and ATT independently of BBB integrity. Age‐related CBF reductions, ATT prolongations, and sex differences in water‐exchange rates can introduce systematic modeling errors, particularly when acquisition parameters are not optimized for diverse hemodynamic states.^191^, ^194^, ^206^, ^207^, ^208^, ^209^, ^210^ Therefore, validation studies must include age‐matched participants, account for sex‐specific physiology, and, when possible, analyze data by sex. All discussed techniques are vulnerable to physiological or pathological factors unrelated to BBB function that alter tissue or vascular properties and therefore affect compartment separation. For multi‐TE ASL, variations in blood oxygenation, hematocrit, and age change blood T2; for DP ASL, FEXI, and WEPCAST, non‐BBB influences on blood velocity (e.g., cardiac function) could affect measured water exchange. Because many such physiological factors remain poorly characterized and cannot yet be reliably modeled or corrected, they pose a major limitation to interpreting water exchange as a BBB biomarker.

Regarding criterion 4, MRI measurements of water exchange operate on 5‐ to 10‐min timescales, making them sensitive to semi‐acute changes in BBB transport but unable to capture rapid transients or clearly distinguish acute from chronic dysfunction. However, because they are fully non‐invasive and require no exogenous tracers, they can be safely repeated for longitudinal monitoring of chronic or progressive BBB degeneration.

In reference to criterion 5, independent validation of water‐exchange MRI techniques remains limited. Comparisons with DCE‐MRI show inconsistent correlations, although this is not surprising given that the methods probe different aspects of BBB transport.^176^, ^177^, ^205^, ^211^ Correlations among water‐exchange techniques themselves are also weak, underscoring limited cross‐method reliability. Consequently, no gold‐standard MRI approach for BBB water exchange currently exists, and more direct method comparisons are needed. Future studies should first identify transport routes within measured timescales and confirm sequence accuracy. Future work should clarify which transport routes are observed within the measured timescales and verify sequence accuracy. Validation in models with known pathology or experimentally modulated BBB disruption, ideally compared with PET, will be essential, though such studies are challenging and costly.

### 5.4 PET

PET imaging is a nuclear imaging technique in which radioactive isotopes are used to evaluate metabolic processes and transport mechanisms in vivo. Targeted radiotracers can be used to detect BBB dysfunction arising from both paracellular permeability and impaired transcellular transport; the principal tracers currently employed are outlined below. For PET imaging, it is important to note that the measured BBB dysfunction reflects exclusively the impairment of the specific transporters for which the selected PET tracers have affinity, or reflects the permeability of these tracers and should not be considered a biomarker for BBB dysfunction in general.

#### 5.4.1 Transport tracers

Efflux transporters at the ECs play an important role in the protection of the brain and maintaining homeostasis by restricting entry of neurotoxic substances into the brain. Among these efflux transporters, P‐gp is the most studied, and changes in P‐gp function are found in several neurodegenerative and psychiatric diseases, as well as in drug–drug interactions. [^11^C]‐verapamil and [^11^C]‐N‐desmethyl‐loperamide are considered the current gold‐standard radiotracers for evaluation of the P‐gp function in vivo.^212^, ^213^, ^214^ These tracers have demonstrated robust reproducibility and sufficient sensitivity to detect disease‐related changes in efflux rate, showing increased P‐gp function in conditions such as epilepsy and schizophrenia and reduced function in AD, PD, and normal aging.^215^, ^216^ However, these tracers show low uptake in the brain at baseline conditions, which complicates the measurement of increases in P‐gp function.^217^ BCRP, another efflux transporter at the BBB, handles the efflux of more than 200 substrates and is also an interesting target for in vivo assessment of BBB dysfunction. However, only a few compounds are reported as potential tracers for BCRP, including dantrolene, tariquidar, and elacridar.^213^ Multidrug resistance protein (MRP) transporters have been assessed using 6‐bromo‐7‐[^11^C]methylpurine and 6‐bromo‐7‐(2‐[^18^F]fluoroethyl)purine, showing promising results in early studies.^218^, ^219^

#### 5.4.2 Glucose transport tracers

[18F]‐fluorodeoxyglucose (FDG) is the most widely PET tracer in general. It is a glucose analog and measures metabolic activity.^217^ The tracer crosses the BBB via the GLUT1 transporter. It accumulates in tissue because it undergoes further metabolism after being phosphorylated to FDG‐6‐phosphate by hexokinase. While it is widely used to quantify cerebral metabolic rate of glucose with dynamic scanning, the FDG influx rate can be estimated by making use of the early tracer signal, when phosphorylation is minimal and uptake is dominated by delivery and BBB transport processes.^220^ Using this method, studies have demonstrated reduced tracer influx in aging, mild cognitive impairment, and early AD.^217^, ^221^, ^222^

#### 5.4.3 Paracellular permeability tracers

Several PET tracers have been proposed as potentially more sensitive alternatives to DCE‐MRI for detecting increased paracellular permeability. Gallium‐based tracers like [^68^Ga] diethylenetriaminepentaacetic acid (DTPA), in general used for evaluation of renal function, are suited for detecting more severe BBB dysfunction due to their relatively high molecular weight (546 Da). In early epileptogenesis, however, this tracer appeared less sensitive than DCE‐MRI, likely due to low tracer concentrations and limited spatial resolution, causing partial volume effects.^223^

Another tracer, [^18^F]2‐Fluoro‐2‐deoxy‐sorbitol (FDS), a sorbitol derivative, showed reproducible sensitivity in detecting BBB permeability after transient BBB opening using focused ultrasound. Because [^18^F]FDS has low BBB permeability under normal conditions and a relatively low molecular weight (182 Da), it serves as a useful baseline for transport studies, although further validation is needed for detecting more subtle dysfunction.^224^ Similarly, 2‐aminoisobutyric acid (AIB), a low‐molecular‐weight (∼103 Da) amino acid easily labeled with ^11^C, has shown promise in detecting increased paracellular permeability in animal models of BBB opening induced by focused ultrasound or lipopolysaccharide.^225^

[^1^ ^5^O]H_2_O (∼17 Da) has been used to assess BBB water transport by comparing its uptake after a single capillary pass to that of freely diffusible [^11^C]butanol (∼74 Da).^226^, ^227^, ^228^ However, its very short half‐life of 2 min requires an on‐site cyclotron, and the associated high costs have limited its use. Another tracer, [^11^C]TGN‐020 (∼208 Da), targets aquaporins to measure water transport, but low spatial resolution limits differentiation between BCSFB transport via aquaporin‐1 and BBB transport via AQP4.^229^, ^230^

In another study, Chung et al. proposed a non‐invasive single‐tracer method to measure the permeability surface area product (PS) of the BBB with the use of three different PET tracers. They demonstrated voxel‐wise measurement of the molecular BBB PS of [^11^C]‐butanol, [^18^F]‐fluciclovine, and [^18^F]‐FDG and obtained promising results by demonstrating PS associations with age in healthy subjects, BBB dysregulation in metabolic dysfunction‐associated steatotic liver disease (MASLD)‐related liver inflammation, and FDG BBB PS associations with blood glucose levels.^231^

#### 5.4.4 Discovering BBB‐dysfunction biomarkers with PET

Regarding criterion 1, PET imaging is by definition a functional imaging technique and provides semi‐quantitative and quantitative measures of several BBB functions, depending on the tracer used. Regarding criterion 2, preferably the PET tracer measures exclusively the functional process of the target of interest, and the signal is not affected by other processes or does not bind to more than one transporter or binding site. At the BBB, P‐glycoprotein and BCRP, for example, have comparable functions and recognize molecules with similar structures, complicating the search for a specific radiotracer that only measures the function of one of these transporters.^232^

Regarding criterion 3, the potential confounders of PET imaging depend on the tracer used and on the chosen outcome parameter. For semi‐quantitative analysis of PET images, the standardized uptake value (SUV) is used, which is obtained from the measured radioactivity values corrected for the injected dose and body weight of the patient.^233^ However, SUV outcomes can be affected by perfusion, non‐specific binding, and the presence of radioactive metabolites. To (partially) overcome these confounders, one can use the SUV ratio, in which the measured values in the organ of interest are set against a reference region.^234^ For full quantification, pharmacokinetic modeling of PET tracers is the gold standard, in which differential equations are used to fit data to several compartment models to describe the influx (K1), efflux (k2), specific binding (BP_ND_), non‐specific binding, and volume of distribution (*V_T_*) of the tracer, reflecting functional biological processes. Additional sources of bias include errors in the arterial input function (e.g., uncorrected delay or dispersion), plasma protein binding, and partial‐volume effects, including spill‐in from nearby blood vessels or CSF spaces and signal loss due to cortical thinning, edema, or atrophy. For glucose transporter tracers, the outcome parameter is additionally modulated by systemic glucose levels, insulin resistance, GLUT expression beyond the endothelium, and secondary neuronal hypometabolism.

Regarding criterion 4, both the temporal resolution of the PET scan and the pharmacokinetics of the tracer should be taken into account. Current clinical dynamic PET has a temporal resolution limited to 5 to 10 s, which is inferior to the temporal resolution of MRI.^235^ PET imaging biomarkers are therefore sufficient to measure semi‐acute and chronic BBB changes but lack temporal resolution for measuring acute changes as well.

Regarding criterion 5, for the measurement of the BBB permeability, the lack of ground truth values of the human BBB in vivo complicates the validation of the aforementioned measurement methods and only indirect approaches can be used and comparisons made with other studies.^231^ For the transporter tracers, the affinity and specificity of the tracers are in general studied in vitro before administration in vivo. Then, indirect validation of the specificity of the radiotracer in vivo is mostly by proof‐of‐concept blocking studies, in which changes in the outcome variables are studied following administration of a specific inhibitor.^236^, ^237^

In conclusion, PET imaging enables targeted assessment of specific BBB transporters and pathways, offering greater molecular specificity than MRI, albeit with lower spatial and temporal resolution, radiation exposure, and higher costs.^180^, ^217^

## 6 DISCUSSION

BBB dysfunction can be assessed using diverse techniques and models, each with unique strengths and limitations (Table 2). Methods are broadly categorized as direct measures (permeability via any of the EC transport pathways) and indirect measures (structural or molecular changes) of BBB dysfunction.

**TABLE 2:** Overview and comparison of BBB dysfunction measurement techniques discussed in this review.

			Spatial scale	Acquisition time	Applicability to A/H	Clinical application	Level of protocol establishment	Cost	Accessibility	Specificity	Direct/indirect measure of BBB dysfunction
**In vitro**		**Paracellular permeability assays**	Paracellular	Minutes to hours	A + H	−	Established	↓	+	++	Direct
		**TEER/ECIS**	Paracellular	Seconds to minutes	A + H	−	Established	↓	+	+	Indirect
		**ICC**	0.02 to 10 µm	N.A.	A + H	−	Established	↓↓	++	+	Indirect
**Ex vivo**	***Ante‐mortem***	**Fluid biomarkers**	Not localized	N.A.	A + H	+ +	Emerging	↓	++	Varies per target	Varies per target
	***Post‐mortem***	**Exogenous tracer distribution**	0.1 to 20 µm	N.A.	A	−	Emerging	↓↓	+	++	Direct
		**IHC/IF**	0.1 to 20 µm	N.A.	A + H	−	Established	↓↓	++	+	Indirect
		**TEM**	1 to 100 nm	N.A.	A + H	−	Established	↑	−	++	Direct + indirect
**In vivo imaging**	**Intravital microscopy**	**Fluorescence imaging**	1 to 20 µm	Seconds to minutes	A	−	Emerging	↓	+	++	Direct + indirect
		**Multiphoton imaging**	1 to 20 µm	Seconds to minutes	A	−	Established	↓	−	++	Direct + indirect
	**MRI**	**DCE‐MRI**	2 mm (H) 0.2 mm (A)	15 to 30 min	A + H	++	Established	↑↑	−	+	Direct
		**ASL**	3 to 5 mm (H) 0.2 to 0.6 mm (A)	7 to 10 min	A + H	+	Emerging	↑↑	−	−	Direct
		**WEPCAST**	Not localized	3 to 5 min	A + H	+	Emerging	↑↑	−	+/−	Direct
		**FEXI**	3 to 5 mm (H) 0.2 to 0.5 (A)	7 to 10 min	A + H	+	Emerging	↑↑	−	−	Direct
	**PET**		3 to 8 mm (H) 0.6 to 2 mm (A)	10 min to 1 h	A + H	+	Emerging	↑↑	−	++	Direct

*Note*: −, low; ± , average; +, high; ++, very high; ↓↓, very low cost; ↓, low cost; ↑, high cost; ↑↑, very high cost.

Abbreviations: A, animals; ASL, arterial spin labeling; BBB, blood–brain barrier; DCE‐MRI, dynamic contrast‐enhanced magnetic resonance imaging; ECIS, electric cell‐substrate impedance sensing; FEXI, filtered‐exchange imaging; H, humans; ICC, immunocytochemistry; IF, immunofluorescence; IHC, immunohistochemistry; N.A., not applicable; PET, positron emission tomography; TEER, trans‐endothelial electrical resistance; TEM, transmission electron microscopy; WEPCAST, Water‐extraction‐with‐phase‐contrast‐arterial‐spin‐tagging.

Studying BBB structure in animals, humans, or in vitro models requires high spatial resolution, achievable using various microscopy techniques. *Post‐mortem* and intravital microscopy provide physiologically relevant insights, with TEM offering the highest resolution but at higher cost and complexity and intravital microscopy enabling real‐time imaging.

Various methods can be used to assess BBB permeability. In vitro models allow controlled investigations of permeability after interventions and in different genetic backgrounds, but lack full complexity. *Post‐mortem* tracer studies provide high‐resolution leakage data but no temporal information. Intravital microscopy gives spatial and temporal resolution but is limited to animals. MRI and PET enable human studies, enhancing clinical translation. DCE‐MRI detects more severe leakage, while water‐exchange MRI captures subtle changes but lacks specificity. PET is able to target various transport pathways, but PET tracers need further validation and optimization. Fluid biomarkers reflect structural and functional BBB changes, with clinical potential that is highly dependent on biomarker quality.

No single method is a gold standard. Integrating multiple approaches provides the most complete understanding of BBB mechanisms in health and disease.

### 6.1 Integrating multiple approaches for BBB research

Integrating complementary techniques strengthens biomarker validation, clarifies their mechanistic or functional relationship with the BBB, reveals links between different BBB markers, and improves translational relevance. The choice of methods depends on the experimental model. In humans, fluid biomarkers, MRI, PET, and *post‐mortem* IHC, IF, and TEM are applicable, whereas rodent studies additionally allow tracer‐based *post‐mortem* approaches and intravital imaging.

Ideally, multiple modalities are combined to comprehensively understand how a biomarker relates to BBB structure and function. In practice, this is often limited by expertise, time, cost, and access to specialized infrastructure. Aligning the research question with the most informative techniques is therefore essential. For example, to investigate whether vascular amyloid plaques in cerebral amyloid angiopathy (CAA) induce BBB dysfunction, in vitro models can first assess whether vascular amyloid increases barrier permeability using tracers. While informative, this approach does not fully resolve the route of leakage, which can be clearly distinguished as paracellular or transcellular using tracer‐based TEM.^114^, ^116^, ^119^ Because in vitro models lack systemic influences, such as immune regulation, vascular remodeling, and clearance via the glymphatic system, in vivo validation is necessary. If in vitro studies suggest that amyloid plaques increase paracellular permeability, permeability in transgenic CAA models should be assessed either after death after intravascular tracer injection or in vivo via preclinical DCE‐MRI, ASL, or PET. Confirming these effects in established CAA models supports translation to human studies, where DCE‐MRI, ASL, or PET can be applied to CAA patients.

### 6.2 Challenges in BBB research

BBB complexity contributes to inconsistencies in its definition. A clear framework for BBB dysfunction as proposed in the introduction improves interpretation, study design, and cross‐study comparisons.

Although techniques assessing BBB permeability are limited, indirect approaches provide valuable insights into underlying biology including possible drug targets to modulate the BBB for clinical applications. Nonetheless, their indirect nature necessitates careful interpretation of results.

Another key challenge in BBB research is the translatability of the various BBB models. Many techniques, including in vitro models, *postmortem* tracers, and intravital microscopy, are restricted to cells or animals, offering high‐resolution insights but not fully capturing human BBB complexity. Additionally, animal models require careful consideration. For instance, AD transgenic mice often represent familial rather than sporadic AD (<5% of AD cases),^238^ and not all models show expected BBB permeability changes (e.g., Tg2576, PS2‐APP,^239^ tau, and APOE ε4 knock‐ins^240^), highlighting the need for careful model selection and cross‐species comparison.

### 6.3 Clinical implications and future directions

The ultimate goal of BBB research is understanding its role in health, disease progression, and therapeutic targeting. Fluid and imaging biomarkers (PET, DCE‐MRI, water‐exchange MRI) may enable early detection and disease monitoring in neurodegeneration, improving interventions and patient understanding of disease trajectory.

Full understanding of BBB structure and function remains incomplete. Future research should optimize imaging, modeling, and biomarker identification, clarify method‐specific measurements, and establish interpretive frameworks.

Promising directions include exploring the influence of BBB dysfunction on neuronal activity, disease progression, and potential therapeutic targets. Combining different research methods, including imaging, fluid biomarkers, and *post mortem* analyses, can yield a comprehensive view of BBB dysfunction dynamics by assessing both structural and functional changes. Cross‐modality integration remains limited, and protocol variability hinders comparability. Standardization and validation across methods are essential.

## CONFLICT OF INTEREST STATEMENT

M.J.P. van Osch receives research support from Philips and serves as a unpaid member of the clinical trial steering committee of the cAPPricorn trial of Alnylam. M.M. Verbeek has an investigator‐initiated research collaboration with Ever Pharma. All other authors have nothing to disclose. Author disclosures are available in the Supporting Information.

## Supporting information



Supporting Information
